# Tumor-associated macrophages promote prostate cancer migration through activation of the CCL22–CCR4 axis

**DOI:** 10.18632/oncotarget.14185

**Published:** 2016-12-26

**Authors:** Aerken Maolake, Kouji Izumi, Kazuyoshi Shigehara, Ariunbold Natsagdorj, Hiroaki Iwamoto, Suguru Kadomoto, Yuta Takezawa, Kazuaki Machioka, Kazutaka Narimoto, Mikio Namiki, Wen-Jye Lin, Guzailinuer Wufuer, Atsushi Mizokami

**Affiliations:** ^1^ Department of Integrative Cancer Therapy and Urology, Kanazawa University Graduate School of Medical Science, Kanazawa, Japan; ^2^ Immunology Research Center, National Health Research Institutes, Zhunan, Miaoli County, Taiwan; ^3^ Hematology Department of The People's Hospital of Xinjiang Uyghur Autonomous Region, Xinjiang, China

**Keywords:** tumor-associated macrophages, CCL2, prostate cancer, CCL22, CCR4

## Abstract

Previous studies have found that tumor-associated macrophages (TAMs) promote cancer progression. We previously reported that TAMs promote prostate cancer metastasis via activation of the CCL2–CCR2 axis. The CCR4 (receptor of CCL17 and CCL22) expression level in breast cancer was reported to be associated with lung metastasis. The aim of this study was to elucidate the role of CCR2 and CCR4 in prostate cancer progression. CCR2 and CCR4 were expressed in human prostate cancer cell lines and prostate cancer tissues. *In vitro* co-culture of prostate cancer cells and macrophages resulted in increased CCL2 and CCR2 levels in prostate cancer cells. The addition of CCL2 induced CCL22 and CCR4 production in prostate cancer cells. The migration and invasion of prostate cancer cells via enhanced phosphorylation of Akt were promoted by CCL17 and CCL22. CCR4 may be a potential candidate for molecular-targeted therapy.

## INTRODUCTION

Prostate cancer represents one of the most frequently diagnosed malignancies in men worldwide [1]. Most patients progress to castration-resistant prostate cancer after several years of androgen-deprivation therapy, and no curative therapies are currently available for this condition [2–4]. Within a tumor microenvironment, chemokines and their receptors are key players in proliferation and metastasis [5, 6]. For instance, CCL2 has been reported to be present in the microenvironment of many types of cancers, and CCR2 has been demonstrated to be upregulated in cancer cells, including prostate cancer cells [7]. We previously reported that tumor-associated macrophages (TAMs) promote prostate cancer metastasis via activation of the CCL2–CCR2 axis [8, 9]. In addition, it has been reported that several chemokines are related to prostate cancer progression [10–12]. CCL17 and CCL22, which are high-affinity ligands of CCR4, have been reported to be secreted by TAMs (M2-type macrophages) and having immunosuppressive functions [13, 14]. Recently, the CCR4 expression level in breast cancer was reported to be associated with lung metastasis [15]. However, the role of CCR4 and the relationship between CCR2 and CCR4 in prostate cancer remains unknown.

Herein, we report that TAMs promote prostate cancer progression through the activation of the CCL2–CCR2 axis, followed by the activation of the CCL17/CCL22–CCR4 axis. *In vitro* co-culture of monocyte-lineage cells with prostate cancer cells induce higher expression of CCL2, which promotes the expressions of CCR2 and CCR4 in prostate cancer cells. Our results suggest that CCR2 and CCR4 play a critical role in prostate cancer progression.

## RESULTS

### Macrophages increased prostate cancer cell migration and invasion

**Figure 1:**
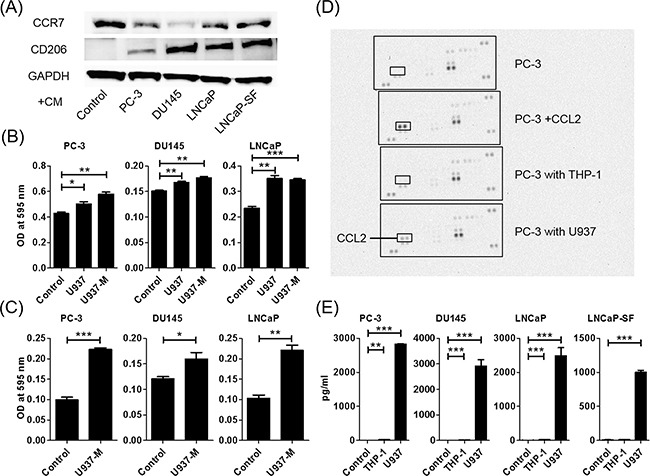
Co-culture of macrophages and prostate cancer cells increases prostate cancer cell migration and invasion and induces CCL2 secretion **A**. U937 differentiation to M2 macrophages is determined using western blot analysis. CCR7 (an M1-type macrophage marker) and CD206 (an M2-type macrophage marker) are assayed using proteins extracted from U937 cells treated with PMA for 24 h and exposed to CM of prostate cancer cells. **B**. Prostate cancer cells are placed in transwell inserts, and CM of U937 and U937-M cells is added. **C**. Prostate cancer cells are placed in transwell inserts with Matrigel^®^-coated membranes in the upper compartment, and U937-M cells are placed in the lower compartments. After 24-h incubation, the cells that had migrated through the membrane are stained. The mean optical density (OD) value is read using a microreader at 595 nm. Data are presented as mean ± SD. **D**. Chemokine arrays comparing the CM of PC-3 cells in monoculture and the CM of PC-3 cells co-cultured with monocyte cells. E. Prostate cancer cells are co-cultured with U937 cells for 24 h, CM is collected, and CCL2 levels are analyzed using ELISA. Adjustments of brightness, contrast, and size are applied to the whole images of western blot-based analyses without elimination of any information present in the original, including backgrounds. The mean OD value is read using a microreader at 450 nm, and data are presented as mean ± SD. All experiments are performed in triplicate, and the mean values are shown. *p < 0.05, **p < 0.01, ***p < 0.001.

U937 cells were used as a model for monocyte-macrophage differentiation because they share many properties with native monocyte-derived macrophages. When floating U937 cells were treated with phorbol 12-myristate 13-acetate (PMA), the cells stopped proliferating, attached to the surface of the plate, and differentiated into macrophages (hereafter termed U937-M) 24 h later. The U937-M cells expressed CCR7, which is an M1-type macrophage marker, but not CD206, which is an M2-type macrophage marker, indicating that U937-M took on M1-type characteristics. After treatment with a conditioned medium (CM) of prostate cancer cells, the U937-M cells expressed CD206, which indicated that the prostate cancer cells could skew macrophages from M1- to M2-type cells, which are almost synonymous with TAMs (Figure 1A). The PC-3, DU145, and LNCaP cells showed significant increases in transwell migration and invasion in response to CM of U937 and U937-M cells (Figures 1B, 1C, and Supplementary Figure 1).

### Macrophages and prostate cancer cells secreted more CCL2 during co-culture

A human cytokine antibody array of CM of PC-3 cells, PC-3 cells treated with CCL2, and PC-3 cells co-cultured with THP-1 and U937 cells showed that co-culturing with U937 cells induced CCL2 secretion and did not induce any other cytokines (Figure 1D). Enzyme-linked immunosorbent assay (ELISA) confirmed that CCL2 secretion was dramatically increased when prostate cancer cells and U937 cells were co-cultured (Figure 1E).

### CCL2 increased migration of prostate cancer cells

**Figure 2:**
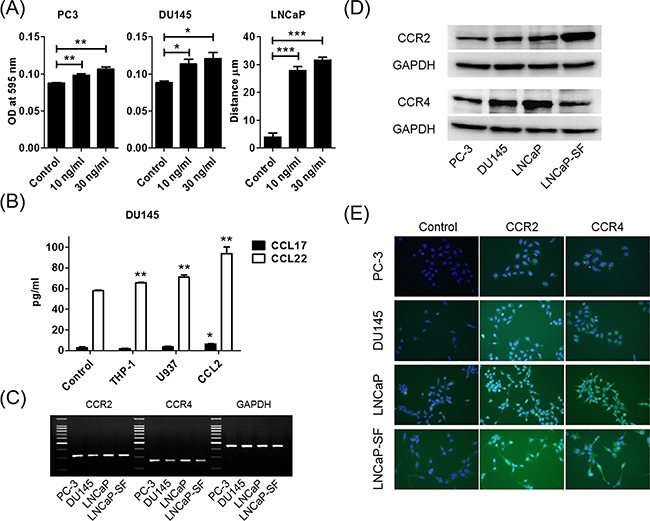
CCL2 promotes prostate cancer cell migration and induces CCL22 secretion, which is a ligand of CCR4 **A**. Prostate cancer cells are placed in transwell inserts and treated with CCL2 (0–30 ng/ml). After 24-h incubation, PC-3 and DU145 cells that had migrated through the membrane are stained. The mean OD value is read using a microreader at 595 nm. Migration of LNCaP cells is assessed with a wound-healing assay. Data are presented as mean ± SD. **B**. DU145 cells are co-cultured with THP-1 and U937 cells and treated with CCL2 (30 ng/ml) for 24 h, CM is collected, and CCL17 and CCL22 levels are analyzed using ELISA. The mean OD value is read using a microreader at 450 nm, and data are presented as mean ± SD. **C, D**. Total RNA and protein are extracted from prostate cancer cells, and CCR2 and CCR4 gene and protein expression levels are analyzed using PCR (C) and western blot (D). **E**. Prostate cancer cells (1.0 × 10^5^ cells/well) are seeded into 6-well plates and cultured until they reach 60%–70% confluence. Cells are incubated with anti-CCR2 or anti-CCR4 antibody and detected using a second antibody conjugated with FITC (green). Cells are counterstained with 4',6-diamidino-2-phenylindole (blue). Adjustments of brightness, contrast, and size are applied to the whole images of western blot-based analyses without elimination of any information present in the original, including backgrounds. All experiments are performed in triplicate, and mean values are shown. *p < 0.05, **p < 0.01, ***p < 0.001.

When prostate cancer cells were treated with human recombinant CCL2, migration was induced in a dose-dependent manner (Figure 2A). As expected, migration of U937 cells was also increased by CCL2 treatment (Supplementary Figure 2). CCL22 and CCL17 were M2-type macrophage markers, and U937-M cells took on the characteristics of M2-type macrophages after co-culture with prostate cancer cells. Therefore, the levels of CCL22 and CCL17 in the co-cultured media of DU145 cells and THP-1 or U937 cells were measured. The CCL22 levels were increased in co-cultured media, but the CCL17 level was very low and could hardly be measured (Figure 2B). Because this finding indicated that CCL2 secretion after co-culture induced CCL22, CCL2 was directly added to DU145 cells and CCL22 was measured. As expected, CCL2 induced CCL22 secretion from DU145 cells (Figure 2B).

### Both CCR2 and CCR4 were expressed in prostate cancer cells

Because CCL2 is a high-affinity ligand for CCR2, CCL2 was thought to increase cell migration through CCR2 receptors. We hypothesized that prostate cancer cells also utilize the CCL2–CCR2 axis and the subsequent CCL17/CCL22–CCR4 axis to metastasize. We first examined CCR2 and CCR4 mRNA expressions in human prostate cancer cells. Quantitative reverse transcription polymerase chain reaction (RT-PCR) showed that CCR2 and CCR4 were expressed in all prostate cancer cell lines that we checked (Figure 2C). Further analyses of protein levels, including western blot and immunocytochemical staining, showed that CCR2 and CCR4 were expressed in all prostate cancer cell lines (Figures 2D and 2E).

### CCL2 increased not only CCL22 but also CCR2 and CCR4

**Figure 3:**
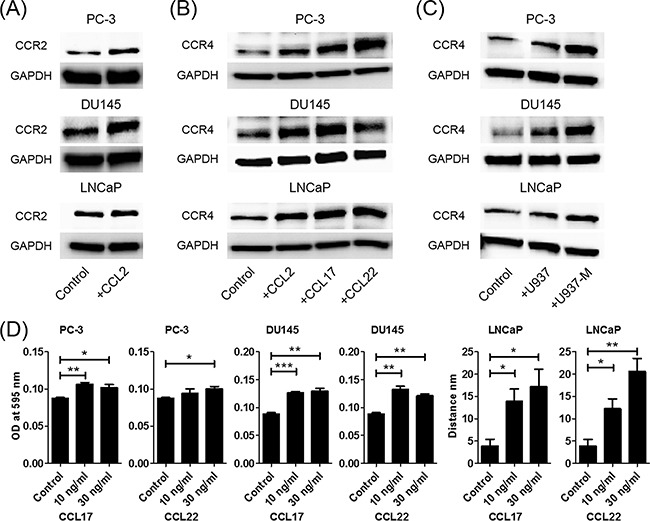
CCL2 increases not only CCL22 but also CCR2 and CCR4 **A**. Prostate cancer cells are treated with CCL2 (30 ng/ml) for 24 h, and western blot for CCR2 is performed. **B**. Prostate cancer cells are treated with CCL2, CCL17, and CCL22 (30 ng/ml) for 24 h, and western blot for CCR4 is performed. **C**. Prostate cancer cells are co-cultured with U937 or U937-M cells for 24 h, and western blot for CCR4 is performed. **D**. Prostate cancer cells are placed in transwell inserts and treated with CCL17 and CCL22 (0–30 ng/ml). After 24-h incubation, PC-3 and DU145 cells that had migrated through the membrane are stained. The mean optical density (OD) value is read using a microreader at 595 nm. Migration of LNCaP cells is assessed with a wound-healing assay. Adjustments of brightness, contrast, and size are applied to the whole images of western blot-based analyses without elimination of any information present in the original, including backgrounds. Data are presented as mean ± SD. All experiments are performed in triplicate, and mean values are shown. *p < 0.05, **p < 0.01, ***p < 0.001.

Previously, it has been reported that co-culture of prostate cancer cells and macrophages increased CCR2 and CCL2 expression levels [8]. To determine whether CCL2 contributes to upregulation of CCR2 in prostate cancer cells, PC-3, DU145, and LNCaP cells were treated with CCL2. Stimulation of prostate cancer cells with CCL2 was found to induce CCR2 production (Figure 3A). Moreover, CCL2, CCL17, and CCL22 stimulation induced CCR4 production (Figure 3B). Consistently, co-culture with U937 and U937-M cells also induced CCR4 production, and the CCR4 production was greater with U937-M cells than with U937 cells (Figure 3C). These results indicated that there is crosstalk between prostate cancer cells and macrophages, and that the CCL2 signal is amplified by CCL22 secretion and upregulation of their receptors.

Next, the effects of CCL17 and CCL22 on prostate cancer cells were analyzed using a transwell migration assay and a wound-healing assay. PC-3 and DU145 cells showed significant increases in migration ability in response to CCL17 and CCL22 treatments (Figure 3D). In the wound-healing assay, CCL17 and CCL22 treatments also significantly enhanced the migration of LNCaP cells (Figure 3D).

### Blockade of the CCL2–CCR2 or CCL17/22–CCR4 axis inhibited the migration of prostate cancer cells

**Figure 4:**
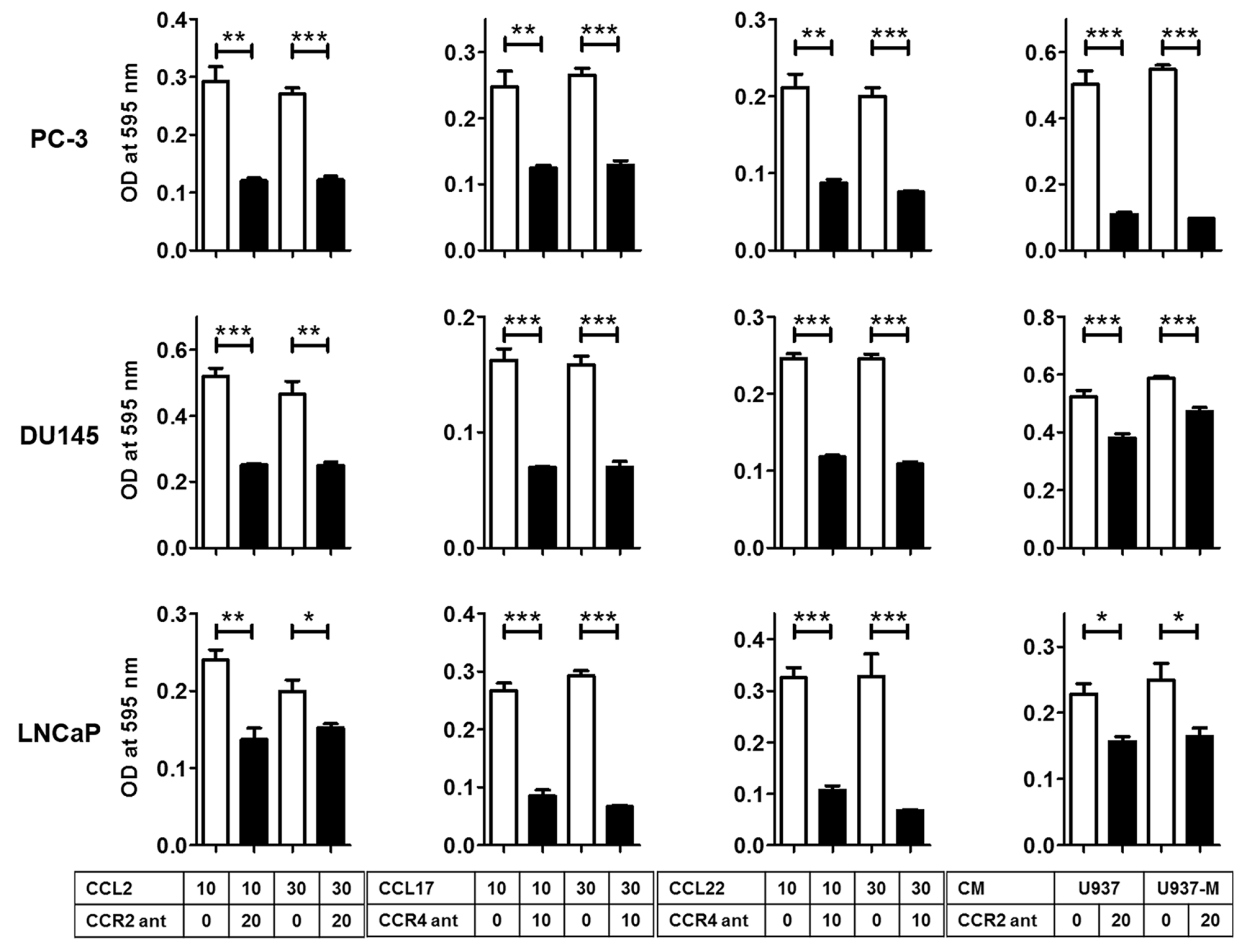
Blockade of the CCL2–CCR2 or CCL17/22–CCR4 axis inhibits the migration of prostate cancer cells Prostate cancer cells are incubated with a CCR2 antagonist (CCR2 ant; 20 μg/ml) or CCR4 antagonist (CCR4 ant; 10 μg/ml) for 30 min and then incubated with CCL2 (10–30 ng/ml), CCL17 (10–30 ng/ml), CCL22 (10–30 ng/ml), or CM of U937 or U937-M cells for 24 h for PC-3 and DU145 and 48 h for LNCaP. The mean optical density (OD) value is read using a microreader at 595 nm. Data are presented as mean ± SD. All experiments are performed in triplicate, and mean values are shown. *p < 0.05, **p < 0.01, ***p < 0.001.

To confirm that the CCL2–CCR2 axis and its downstream CCL17/22–CCR4 axis really contributed to the induction of prostate cancer cell migration, the CCL2–CCR2 or CCL17/22–CCR4 axis was blocked using a receptor antagonist. CCR2 and CCR4 antagonists clearly inhibited the migration ability of prostate cancer cells, which was induced by CCL2 and CCL17/CCL22 (Figure 4). Interestingly, a CCR4 antagonist could inhibit the migration ability induced by CCL2 (Supplementary Figure 3A). Moreover, a CCR2 antagonist also inhibited the migration ability of prostate cancer cells induced by U937 and U937-M cells (Figure 4). As expected, increased migration of U937 cells caused by CCL2 treatment was also inhibited by the CCR2 antagonist (Supplementary Figure 3B). These results showed that CCR2 and CCR4 expressed by the prostate cancer cells were sufficient to cause migration when treated with CCL2, CCL17, and CCL22, or CM collected from U937 cells and M2-type macrophages U937-M cells.

### CCR2 and CCR4 were expressed more strongly in prostate cancer tissue than in normal prostate tissue

**Figure 5:**
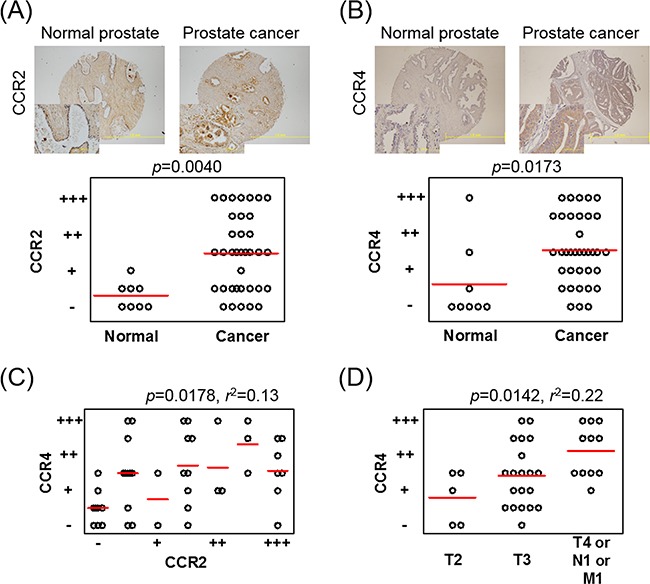
CCR4 and CCR2 are increased in prostate cancer tissues on IHC staining **A, B**. A TMA plate containing tissue samples from 8 patients with normal prostates and tissue samples from 36 patients with prostate cancer is used, and one or two spotted tissues from each patient are placed on the plate. IHC staining of CCR2 (A) and CCR4 (B) is performed and scored on a scale from – to +++, according to intensity. Patients having two spotted tissues are scored using the average value. **C**. Correlation between the staining intensities of CCR2 and CCR4 in each patient is analyzed using Pearson's correlation coefficient test. **D**. The CCR4 staining intensity is analyzed according to the progression of TNM stage. One-way ANOVA is performed. Bars indicate the mean of each group.

To determine CCR2 and CCR4 receptor expressions in human prostate cancer tissue, immunohistochemical (IHC) staining was performed using a pair of the same tissue microarray (TMA) plates. The backgrounds of the patients are shown in Supplementary Table 1. IHC analysis revealed a significant increase in both CCR2 and CCR4 expressions in prostate cancer cells compared with the expressions in epithelial cells in normal prostate tissues (Figures 5A and 5B). Interestingly, the staining intensities of CCR2 and CCR4 in each specimen were significantly correlated (Supplementary Figure 4A). When the staining intensities of tissues were averaged in every patient, the staining intensities of CCR2 and CCR4 still showed significant correlations (Figure 5C). The staining intensity of CCR2 was not correlated with the progression of TNM stage (Supplementary Figure 4B), whereas that of CCR4 was correlated (Figure 5D). The staining intensities of CCR2 and CCR4 were not correlated with the Gleason score (Supplementary Figures 4C and 4D). These results from clinical samples support our *in vitro* data showing that the CCL2–CCR2 axis-induced CCL22 triggered CCR4 production and that both the CCL2–CCR2 and CCL22–CCR4 axes contributed to prostate cancer progression.

### Activation of Akt is a critical step downstream of the CCL22–CCR4 axis for prostate cancer migration

**Figure 6:**
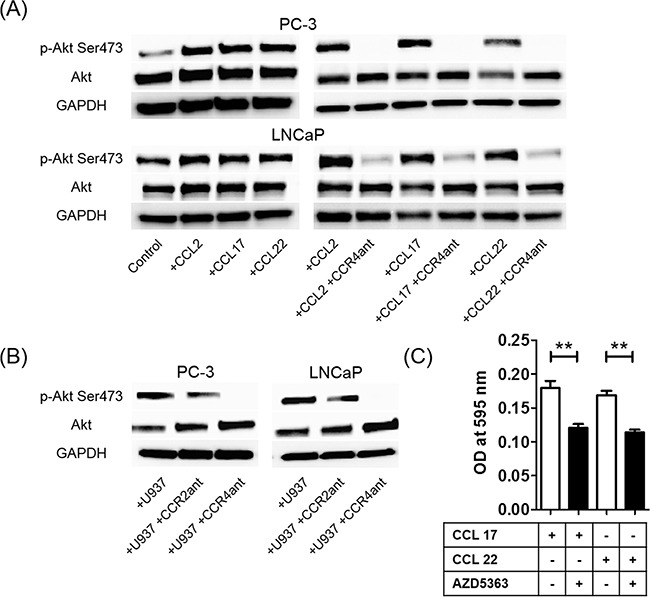
The CCL2–CCR2 and CCL22–CCR4 axes increase phosphorylation of Akt **A**. Phosphorylation of Akt (Ser473) proteins in prostate cancer cells is assayed using western blot at 30 min after CCL2, CCL17, or CCL22 stimulation (left panels) and after CCR4 antagonist treatment with CCL2, CCL17, or CCL22 stimulation (right panels). **B**. U937 cell-induced phosphorylation of Akt (Ser473) proteins in prostate cancer cells with or without CCR2 and CCR4 antagonists is also assayed using western blot. Adjustments of brightness, contrast, and size are applied to the whole images of western blot-based analyses without elimination of any information present in the original, including backgrounds. **C**. PC-3 cells are incubated with the Akt inhibitor AZD5363 (10 μg/ml) with CCL17 (30 ng/ml) and CCL22 (30 ng/ml) for 24 h. The mean optical density (OD) value is read using a microreader at 595 nm. Data are presented as mean ± SD. All experiments are performed in triplicate, and mean values are shown. **p < 0.01. All experiments are performed in triplicate, and representative data are shown.

To verify that CCR2 and CCR4 are functional in prostate cancer cells, the effects of CCL2, CCL17, and CCL22 on the activation of Akt signaling pathways were tested. Rapid phosphorylation of Akt (Ser473) was observed at 30 min after CCL2, CCL17, and CCL22 treatment (Figure 6A), but not Thr308 (Supplementary Figure 5). A CCR4 antagonist strongly inhibited chemokine-induced phosphorylation of Akt (Ser473) (Figure 6A), and a CCR2 antagonist strongly inhibited CCL2-induced phosphorylation of Akt (Ser473) (Supplementary Figure 6). U937 cell-induced phosphorylation of Akt (Ser473) was also inhibited by the CCR2 and CCR4 antagonists (Figure 6B). Because the phosphorylation of Akt (Ser473) was more strongly inhibited by the CCR4 antagonist than by the CCR2 antagonist, CCR4 may contribute more to prostate cancer cell migration than CCR2 (Figure 6B). Moreover, AZD5363, a potent inhibitor of Akt, inhibited CCL17- and CCL22-induced prostate cancer migration (Figure 6C). These findings showed that TAMs phosphorylated Akt and induced prostate cancer cell migration and invasion via activation of the CCL22–CCR4 axis.

## DISCUSSION

It has been established that the majority of malignant tumors contain numerous macrophages as major components of the host leukocytic infiltrate [16]. Macrophage recruitment is mediated by a variety of chemoattractants, and macrophages in tumor microenvironments are defined as TAMs and exhibit an M2-type characteristic [17]. It has been shown that CCL2 plays an important role in macrophage accumulation at the tumor site through evidence indicating that tumor-derived CCL2 levels were correlated with the abundance of TAMs in several types of cancers [18–21]. TAMs have been reported to secrete M2-type macrophage markers, including both CCL17 and CCL22; therefore, our study focused on the CCL17/CCL22–CCR4 axis with regard to interactions between TAMs and prostate cancer cells. However, to our knowledge, the expression and role of CCR4 in prostate cancer have not been reported so far, and little is known about the relationship between TAMs and the CCL2–CCR2 and CCL17/CCL22–CCR4 axes with regard to tumor migration and invasion in prostate cancer. We hypothesized that prostate cancer cells utilize the CCL2–CCR2 and CCL17/CCL22–CCR4 axes to metastasize through interactions between TAMs and prostate cancer cells.

**Figure 7:**
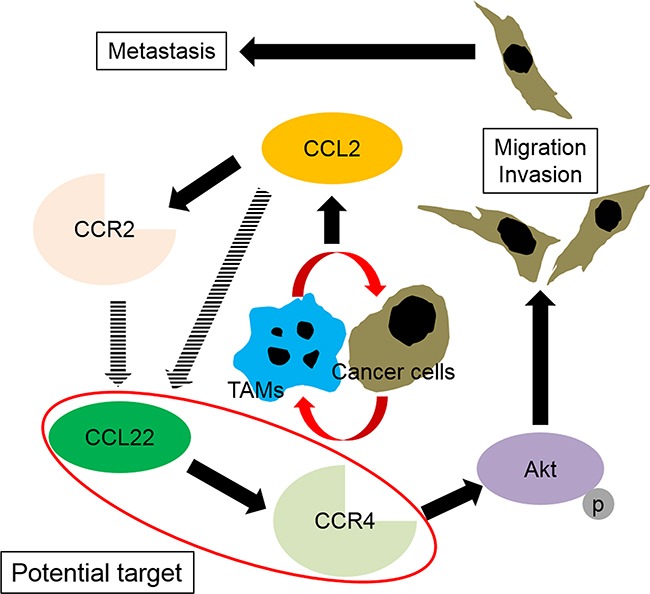
Schematic illustration of the spiral effects of the CCL2–CCR2 and CCL22–CCR4 axes through interactions between tumor-associated macrophages (TAMs) and prostate cancer cells Infiltrated macrophages become M2-type cells (TAMs) through stimulation with prostate cancer cells. The interactions between TAMs and prostate cancer cells induce CCL2 in prostate cancer cells and affect the TAMs and prostate cancer cells in an autocrine manner. CCL2 upregulates its receptor, and through acceleration of the CCL2–CCR2 signal, CCL22 and CCR4 are upregulated. Subsequently, the CCL22–CCR4 axis activates Akt, which results in prostate cancer migration and invasion.

We demonstrated the CCR2 and CCR4 gene and protein expressions in prostate cancer cells as well as in human prostate cancer tissues, which are novel findings for CCR4. Interestingly, CCL2 treatment resulted in significant increases in CCR2 and CCR4 protein levels in prostate cancer cells and promoted recruitment of monocytes into the tumor microenvironment, where they differentiated into TAMs. Inhibition of the CCL2–CCR2 or CCL17/CCL22–CCR4 axis by specific antagonists of CCR2 or CCR4 clearly showed suppression of the migration ability of prostate cancer cells. Because the TMA analysis showed that the staining intensity of CCR4 correlated with prostate cancer progression, while that of CCR2 did not, despite the fact that their intensities were correlated with each other, CCR4 may be a more powerful driver of prostate cancer migration and invasion than CCR2. The fact that phosphorylation of Akt proteins was more effectively inhibited by the CCR4 antagonist than by the CCR2 antagonist also indicates the efficiency of CCR4 in prostate cancer migration and invasion. Akt activation is controlled by phosphorylation of the two key residues threonine 308 (Thr308) and serine 473 (Ser473) [22], and their phosphorylation promotes prostate cancer cell growth, proliferation, motility, and survival [23–25]. Our results indicated that the CCL22–CCR4 axis controlled phosphorylation of Ser473. It has been previously demonstrated that CCL2 promotes prostate cancer cell proliferation, migration, and survival via Akt-activation-dependent mechanisms [26–28]. These results indicate that the CCL22–CCR4 axis is a better potential target than the CCL2–CCR2 axis for the future treatment of patients with prostate cancer (Figure 7).

Furthermore, the CCL22–CCR4 axis may be a novel biomarker for prostate cancer. The limitations of using a prostate-specific antigen alone as a biomarker for prostate cancer have been reported, and the necessity of more specific and effective biomarkers has been suggested [29, 30]. Previously, we reported that the serum CCL2 level in patients with prostate cancer was clearly predictive of the time taken by the tumor to become castration resistant and of overall and prostate cancer-specific survivals [31]. However, additional clinical evidence is needed to show the superiority of the CCL22–CCR4 axis over the CCL2–CCR2 axis as a prostate cancer biomarker.

## MATERIALS AND METHODS

### Reagents and antibodies

Recombinant human CCL17 and CCL22 were purchased from R&D Systems (Minneapolis, MN). Recombinant human CCL2 was obtained from BioLegend (San Diego, CA). Antibodies used in western blotting, IHC, immunocytochemistry, and flow cytometry were as follows: rabbit anti-human CCR2 antibody (ab21667), rabbit anti-human CCR4 antibody (ab83250), goat anti-human CCR4 antibody (ab1669), rabbit anti-goat immunoglobulin (Ig)G H&L (fluorescein isothiocyanate; FITC) antibody (6737), goat anti-rabbit IgG H&L (FITC) antibody (6717), anti-mannose receptor antibody (ab64693), and anti-CCR7 antibody (ab103404) obtained from Abcam (Cambridge, MA); anti-Akt antibody (9272), anti-phospho-Akt (Thr308) antibody (9275), anti-phospho-Akt (Ser473) antibody (9271), and anti-rabbit IgG HRP-linked antibody (7074) obtained from Cell Signaling Technology (Danvers, MA); and anti-GAPDH antibody (NB300-221) obtained from Novus Biologicals (Littleton, CO). The antagonists used in the neutralizing assay were a selective CCR2 antagonist (ab120812) obtained from Abcam and a CCR4 antagonist (SC-221406) obtained from Santa Cruz Biotechnology (Dallas, TX). The Akt inhibitor AZD5363 (S8019) was obtained from Selleckchem (Houston, TX).

### Cell culture

The human prostate cancer cell lines PC-3, DU145, and LNCaP were purchased from American Type Culture Collection (Manassas, VA). The human monocytoid cell lines U937 and THP-1 were purchased from HPA Culture Collections and Public Health England (Salisbury, UK). Prostate cancer cells were maintained in a humidified atmosphere of 5% CO_2_ at 37°C in Roswell Park Memorial Institute (RPMI) 1640 medium supplemented with 5% fetal bovine serum (FBS) and 1% penicillin/streptomycin (Thermo Fisher Scientific, Waltham, MA). U937 and THP-1 cells were maintained in a humidified atmosphere of 5% CO_2_ at 37°C in RPMI 1640 medium supplemented with 10% FBS and 1% penicillin/streptomycin. An androgen-independent LNCaP cell line (LNCaP-SF) was established in our laboratory after long-term subculture of the parental LNCaP cells in Dulbecco's modified Eagle's medium supplemented with 5% charcoal-stripped FBS (CCS) and 1% penicillin/streptomycin.

### Macrophage differentiation

M2-polarized U937 macrophages were generated as shown below. First, 20 ml of U937 cells (2.5 × 10^5^ cells/ml) were treated with 2 μl of PMA (100 ng/ml) and were then seeded into 6-well plates. Cells were incubated for 24 h in a humidified atmosphere of 5% CO_2_ at 37°C to differentiate into macrophages and then washed three times with PBS to remove PMA. Subsequently, the cells were exposed to CM of prostate cancer cells for 4 days.

### Co-culture assay

The co-culture experiments were performed using transwell inserts in 6-well plates (cell culture inserts with 1.0-μm pore sizes for 6-well plates; Greiner Bio-One, Monroe, NC). Briefly, PC-3, DU145, LNCaP, and LNCaP-SF cells (1.0 × 10^5^ cells) in the appropriate medium were placed into the upper compartment, and the lower compartment was filled with RPMI-1640 containing 5% FBS or 5% CCS. Cells were allowed to attach for 4 h, and then, U937 cells (2.0 × 10^5^ cells) or M2-polarized U937 macrophages were added to the lower compartment and co-cultured for 24 or 48 h.

### RT-PCR analysis

Total RNA was isolated from the cultured cells using the RNeasy Mini Kit (Qiagen, Germantown, MD), following the manufacturer's instructions. The RNA concentrations of the cell lines were measured using a NanoDrop spectrophotometer (Thermo Fisher Scientific). First-strand cDNA was prepared from an RNA template (500 ng) using the iScript cDNA Synthesis Kit (Bio-Rad, Hercules, CA). Reverse transcription was performed at 25°C for 5 min, 42°C for 30 min, and then 85°C for 5 min. PCR amplification for glyceraldehyde-3-phosphate dehydrogenase (GAPDH) (98°C for 10 s, 60°C for 30 s, 72°C for 60 s, 26 cycles), CCR2, and CCR4 (98°C for 10 s, 60°C for 30 s, 72°C for 60 s, 35 cycles) was performed using template cDNA and the TaKaRa Ex Taq Hot Start Version PCR kit (Takara Bio, Kusatsu, Japan). PCR products were electrophoresed on 1.5% agarose gels and stained with ethidium bromide. The sequences of the primers are shown in Supplementary Table 2.

### Western blot analysis

Cell lysates were prepared with M-PER Mammalian Protein Extraction Reagent (Thermo Fisher Scientific). The soluble lysate (30 μg) was mixed with LDS sample buffer and sample reducing agent and was resolved with sodium dodecyl sulfate-polyacrylamide gel electrophoresis. The proteins were transferred to nitrocellulose membranes. The membranes were blocked with 1% gelatin in 0.05% Tween in Tris-buffered saline for 1 h at room temperature (RT) and then incubated overnight at 4°C with primary antibodies as indicated. CCR2, CCR4, Akt, and phospho-Akt (Ser473) were detected using antibodies as described by the manufacturer. Membranes were washed three times before incubation with HRP-conjugated anti-rabbit secondary antibodies for 1 h at RT. Protein loading was determined with an anti-GAPDH antibody to detect levels of GAPDH. Protein bands were detected using SuperSignal West Femto Maximum Sensitivity Substrate (Thermo Fisher Scientific). For analyses of upregulation of CCR4 and CCR2 in prostate cancer cells, the cells were seeded in 6-well plates and allowed to reach 60%–70% confluence, followed by incubation with recombinant human CCL2, CCL17, and CCL22 (30 ng/ml) for 24 h.

### Immunocytochemical staining

The prostate cancer cells (1.0 × 10^5^ cells/well) were seeded into 6-well plates and cultured until they reached 60%–70% confluence. The cells were washed with PBS and fixed in 4% paraformaldehyde in PBS for 15 min at RT, and they were then washed three times with cold PBS before permeabilization with 0.25% Triton X-100 in PBS for 15 min at RT. The cells were washed three times with PBS and blocked with 1% bovine serum albumin (BSA) in polybutylene succinate-co-butylene terephthalate at RT for 30 min, and they were then incubated with a primary antibody overnight at 4°C, as described by the manufacturer. CCR2 and CCR4 were detected by incubating the cells for 1 h with a secondary antibody conjugated with FITC (1:1000). The cells were counterstained with 4', 6-diamidino-2-phenylindole (Sigma-Aldrich, St. Louis, MO). Images were taken using Olympus FSX100 (Olympus, Tokyo, Japan).

### Tissue microarray (TMA) analysis

A human prostate cancer TMA (PR 956a) was purchased from US Biomax (Rockville, MD). A TMA plate contains tissue samples from 8 patients with normal prostates and 36 patients with prostate cancer, and one or two spotted tissues of each patient are placed on the array. IHC staining was performed using the EnVision Detection System Peroxidase/DAB+ kit (K5007) according to the manufacturer's protocol (Dako, Glostrup, Denmark) and the method described by Shin et al. [32]. Tissue specimens were stained with rabbit polyclonal antibody against CCR2 and goat polyclonal antibody against CCR4. Microwave antigen retrieval was performed in Dako Target Retrieval Solution for 10 min. The arrays were analyzed by a blinded pathologist, and the intensities of the stained epithelial cells were recorded. Staining intensity was scored on a scale from – to +++ (–, negative; +, weak; ++, moderate; +++, strong intensity staining).

### Wound-healing assay

LNCaP cells (1.0 × 10^6^ cells/well) were seeded into 6-well plates. After reaching full confluence, the monolayer of cells was scratched with a 100-μl sterile pipette tip to make a vertical wound. To avoid the influence of the cell growth rate on wound healing, the tumor cell culture medium was changed from RPMI 1640 medium supplemented with 5% FBS to RPMI 1640 medium supplemented with 0.2% FBS and 0.2% BSA or different concentrations of CCL2, CCL17, or CCL22. Phase-contrast images were taken of each sample at 0 and 24 h with a 10× objective using Olympus FSX100. Migration activity was expressed by the transferred distance method measured using ImageJ (National Institutes of Health, Bethesda, MD) and calculated by subtraction of the distances at 0 h and 24 h. Three separate visual fields were measured in each experiment. Statistical analysis was performed using Student's *t*-test. A *p*-value < 0.05 was considered statistically significant.

### Cell migration and invasion assay

The migration assay was performed using transwell inserts placed in a 24-well plate (cell culture inserts for 24-well multiwell plates with 8.0-μm pore sizes; Greiner Bio-One). For the invasion assay, the transwell inserts were coated with 100 μl of 1:5 diluted Matrigel^®^ (Corning, Corning, NY) in an atmosphere of 5% CO_2_ at 37°C for 5 h, and then, serum-free medium was added to the interior of the bottom compartment and to the upper compartment of the inserts and allowed to hydrate in an atmosphere of 5% CO_2_ at 37°C for 2 h. Prostate cancer cells (PC-3, DU145) were grown to 80% confluence in an appropriate medium. The cells were synchronized by starvation in serum-free RPMI 1640 containing 0.5% BSA in a humidified atmosphere of 5% CO_2_ at 37°C for 16 h. Approximately 2.5 × 10^4^ cells in 200 μl of RPMI-1640 culture media supplemented with 0.1% FBS were placed in the upper compartment, and the lower compartment was filled with 600 μl of RPMI-1640 containing 2% FBS. The cells were allowed to attach for 4 h, and then, the lower compartment was changed to 600 μl of RPMI-1640 containing 2% FBS with or without different concentrations of CCL2, CCL17, CCL22, and/or CM of U937 or M2-polarized U937 macrophages. The cancer cell invasion assay was cultured with or without 5.0 × 10^4^ M2-polarized U937 macrophages added to the lower compartment. The cells were incubated at 37°C in an atmosphere of 5% CO_2_ for 20 h and fixed in 4% paraformaldehyde in PBS for 10 min. The cells on the upper surface of the filter were removed carefully with a cotton swab. The cells on the lower side of the filter were stained with 0.1% crystal violet for 15 min, and the stained filters were photographed. The crystal violet dye retained on the filters was extracted into 33% acetic acid. Cell migration was measured by reading the absorbance at 590 nm and was corrected at 450 nm on a microplate reader. Statistical analysis was performed using Student's *t*-test.

For the CCR2 and CCR4 neutralizing experiments, cancer cells were incubated with CCR2 or CCR4 antagonist at a concentration ranging from 10 μg/ml to 20 μg/ml in RPMI-1640 culture media supplemented with 0.1% FBS at RT for 30 min and then seeded into the upper compartment. The lower compartment was filled with 600 μl of RPMI-1640 containing 2% FBS with or without different concentrations of CCL2, CCL17, CCL22, and/or CM from U937 or M2-polarized U937 macrophages. Cells were incubated in 5% CO_2_ at 37°C for 24 h (PC-3 and DU145) or 48 h (LNCaP).

### Human cytokine antibody array

CM was collected from PC-3 cells (control), PC-3 cells treated with CCL2 for 24 h, and PC-3 cells co-cultured with monocyte cell lines, THP-1, and U937 cells. Cytokine levels were determined using a Human Cytokine Array kit (ARY017, R&D systems) according to the manufacturer's instructions.

### Enzyme-linked immunosorbent assay (ELISA) assay

Human CCL2 secretion was measured in serum-free CM from PC-3, DU145, LNCaP, LNCaP-SF, and co-cultured media with U937 or macrophages using a Quantikine human ELISA kit purchased from R&D systems, according to the manufacturer's instructions. Absorbance was measured at 450 nm and was corrected at 540 nm on a microplate reader. Total protein concentrations of the CM were calculated using CurveExpert 1.4. (CurveExpert and GraphExpert Software, Madison, AL). Statistical analysis was performed using Student's *t*-test. A *p*-value < 0.05 was considered statistically significant.

### Conditioned medium (CM)

For preparing the prostate cancer cell CM, 1.0 × 10^6^ prostate cancer cells were seeded into 6-well culture plates and allowed to adhere overnight. On the next day, the medium was aspirated and 2 ml of medium containing 1% FBS was added, and the supernatant was collected 48 h later. The supernatant was centrifuged at 1800 rpm for 10 min and was collected as CM.

## CONCLUSION

To our knowledge, this is the first study to show that CCR4 was expressed in prostate cancer cell lines and human prostate cancer tissues and that the CCL22–CCR4 axis contributed to prostate cancer migration and invasion. The CCL22–CCR4 axis, which is activated by TAMs, may be a novel therapeutic target and a potential biomarker for prostate cancer.

## SUPPLEMENTARY FIGURES AND TABLES



## Abbreviations

TAMs: tumor-associated macrophages
ELISA: enzyme-linked immunosorbent assay
PMA: phorbol 12-myristate 13-acetate
CM: conditioned medium
RT-PCR: reverse transcription polymerase chain reaction
IHC: immunohistochemical
TMA: tissue microarray
FBS: fetal bovine serum
CCS: charcoal-stripped FBS
GAPDH: glyceraldehyde-3-phosphate dehydrogenase
RT: room temperature
BSA: bovine serum albumin

## References

[1] Siegel RL, Miller KD, Jemal A (2016). Cancer statistics, 2016. CA Cancer J Clin.

[2] Denis L (1993). Prostate cancer. Primary hormonal treatment. Cancer.

[3] Izumi K, Namiki M (2014). Optimal treatment for castration-resistant prostate cancer. Asian J Androl.

[4] Shah RB, Mehra R, Chinnaiyan AM, Shen R, Ghosh D, Zhou M, Macvicar GR, Varambally S, Harwood J, Bismar TA, Kim R, Rubin MA, Pienta KJ (2004). Androgen-independent prostate cancer is a heterogeneous group of diseases: lessons from a rapid autopsy program. Cancer Res.

[5] Tanaka T, Bai Z, Srinoulprasert Y, Yang BG, Hayasaka H, Miyasaka M (2005). Chemokines in tumor progression and metastasis. Cancer Sci.

[6] Vicari AP, Caux C (2002). Chemokines in cancer. Cytokine Growth Factor Rev.

[7] Lu Y, Cai Z, Xiao G, Liu Y, Keller ET, Yao Z, Zhang J (2007). CCR2 expression correlates with prostate cancer progression. J Cell Biochem.

[8] Izumi K, Fang LY, Mizokami A, Namiki M, Li L, Lin WJ, Chang C (2013). Targeting the androgen receptor with siRNA promotes prostate cancer metastasis through enhanced macrophage recruitment via CCL2/CCR2-induced STAT3 activation. EMBO Mol Med.

[9] Lin TH, Izumi K, Lee SO, Lin WJ, Yeh S, Chang C (2013). Anti-androgen receptor ASC-J9 versus anti-androgens MDV3100 (Enzalutamide) or Casodex (Bicalutamide) leads to opposite effects on prostate cancer metastasis via differential modulation of macrophage infiltration and STAT3-CCL2 signaling. Cell Death Dis.

[10] Fang LY, Izumi K, Lai KP, Liang L, Li L, Miyamoto H, Lin WJ, Chang C (2013). Infiltrating macrophages promote prostate tumorigenesis via modulating androgen receptor-mediated CCL4-STAT3 signaling. Cancer Res.

[11] Izumi K, Chang C (2013). Targeting inflammatory cytokines-androgen receptor (AR) signaling with ASC-J9 to better battle prostate cancer progression. Oncoimmunology.

[12] Izumi K, Li L, Chang C (2014). Androgen receptor and immune inflammation in benign prostatic hyperplasia and prostate cancer. Clin Investig (Lond).

[13] Hefetz-Sela S, Stein I, Klieger Y, Porat R, Sade-Feldman M, Zreik F, Nagler A, Pappo O, Quagliata L, Dazert E, Eferl R, Terracciano L, Wagner EF (2014). Acquisition of an immunosuppressive protumorigenic macrophage phenotype depending on c-Jun phosphorylation. Proc Natl Acad Sci U S A.

[14] Mantovani A, Sozzani S, Locati M, Allavena P, Sica A (2002). Macrophage polarization: tumor-associated macrophages as a paradigm for polarized M2 mononuclear phagocytes. Trends Immunol.

[15] Olkhanud PB, Baatar D, Bodogai M, Hakim F, Gress R, Anderson RL, Deng J, Xu M, Briest S, Biragyn A (2009). Breast cancer lung metastasis requires expression of chemokine receptor CCR4 and regulatory T cells. Cancer Res.

[16] van Ravenswaay Claasen HH, Kluin PM, Fleuren GJ (1992). Tumor infiltrating cells in human cancer. On the possible role of CD16+ macrophages in antitumor cytotoxicity. Lab Invest.

[17] Sica A, Schioppa T, Mantovani A, Allavena P (2006). Tumour-associated macrophages are a distinct M2 polarised population promoting tumour progression: potential targets of anti-cancer therapy. Eur J Cancer.

[18] Monti P, Leone BE, Marchesi F, Balzano G, Zerbi A, Scaltrini F, Pasquali C, Calori G, Pessi F, Sperti C, V Di Carlo, Allavena P, Piemonti L (2003). The CC chemokine MCP-1/CCL2 in pancreatic cancer progression: regulation of expression and potential mechanisms of antimalignant activity. Cancer Res.

[19] Negus RP, Stamp GW, Relf MG, Burke F, Malik ST, Bernasconi S, Allavena P, Sozzani S, Mantovani A, Balkwill FR (1995). The detection and localization of monocyte chemoattractant protein-1 (MCP-1) in human ovarian cancer. J Clin Invest.

[20] Nesbit M, Schaider H, Miller TH, Herlyn M (2001). Low-level monocyte chemoattractant protein-1 stimulation of monocytes leads to tumor formation in nontumorigenic melanoma cells. J Immunol.

[21] Ueno T, Toi M, Saji H, Muta M, Bando H, Kuroi K, Koike M, Inadera H, Matsushima K (2000). Significance of macrophage chemoattractant protein-1 in macrophage recruitment, angiogenesis, and survival in human breast cancer. Clin Cancer Res.

[22] Manning BD, Cantley LC (2007). AKT/PKB signaling: navigating downstream. Cell.

[23] Assinder SJ, Dong Q, Kovacevic Z, Richardson DR (2009). The TGF-beta, PI3K/Akt and PTEN pathways: established and proposed biochemical integration in prostate cancer. Biochem J.

[24] Majumder PK, Sellers WR (2005). Akt-regulated pathways in prostate cancer. Oncogene.

[25] New DC, Wu K, Kwok AW, Wong YH (2007). G protein-coupled receptor-induced Akt activity in cellular proliferation and apoptosis. FEBS J.

[26] Loberg RD, Day LL, Harwood J, Ying C, St John LN, Giles R, Neeley CK, Pienta KJ (2006). CCL2 is a potent regulator of prostate cancer cell migration and proliferation. Neoplasia.

[27] Loberg RD, Ying C, Craig M, Day LL, Sargent E, Neeley C, Wojno K, Snyder LA, Yan L, Pienta KJ (2007). Targeting CCL2 with systemic delivery of neutralizing antibodies induces prostate cancer tumor regression in vivo. Cancer Res.

[28] Roca H, Varsos Z, Pienta KJ (2008). CCL2 protects prostate cancer PC3 cells from autophagic death via phosphatidylinositol 3-kinase/AKT-dependent survivin up-regulation. J Biol Chem.

[29] Izumi K, Lin WJ, Miyamoto H, Huang CK, Maolake A, Kitagawa Y, Kadono Y, Konaka H, Mizokami A, Namiki M (2014). Outcomes and predictive factors of prostate cancer patients with extremely high prostate-specific antigen level. J Cancer Res Clin Oncol.

[30] Izumi K, Ikeda H, Maolake A, Machioka K, Nohara T, Narimoto K, Ueno S, Kadono Y, Kitagawa Y, Konaka H, Mizokami A, Namiki M (2015). The relationship between prostate-specific antigen and TNM classification or Gleason score in prostate cancer patients with low prostate-specific antigen levels. Prostate.

[31] Izumi K, Mizokami A, Lin HP, Ho HM, Iwamoto H, Maolake A, Natsagdorj A, Kitagawa Y, Kadono Y, Miyamoto H, Huang CK, Namiki M, Lin WJ (2016). Serum chemokine (CC motif) ligand 2 level as a diagnostic, predictive, and prognostic biomarker for prostate cancer. Oncotarget.

[32] Shin M, Mizokami A, Kim J, Ofude M, Konaka H, Kadono Y, Kitagawa Y, Miwa S, Kumaki M, Keller ET, Namiki M (2013). Exogenous SPARC suppresses proliferation and migration of prostate cancer by interacting with integrin beta1. Prostate.

