# Supplementary material for: Tumor-associated macrophages promote prostate cancer migration through activation of the CCL22–CCR4 axis
Source: Oncotarget. 2016 Dec 26;8(6):9739–51. doi: 10.18632/oncotarget.14185 (PMC5354767; doi:10.18632/oncotarget.14185)
Supplement: Supplementary file 1 [file oncotarget-08-9739-s001.pdf]

# Tumor-associated macrophages promote prostate cancer migration through activation of the CCL22–CCR4 axis

## SUPPLEMENTARY FIGURES AND TABLES

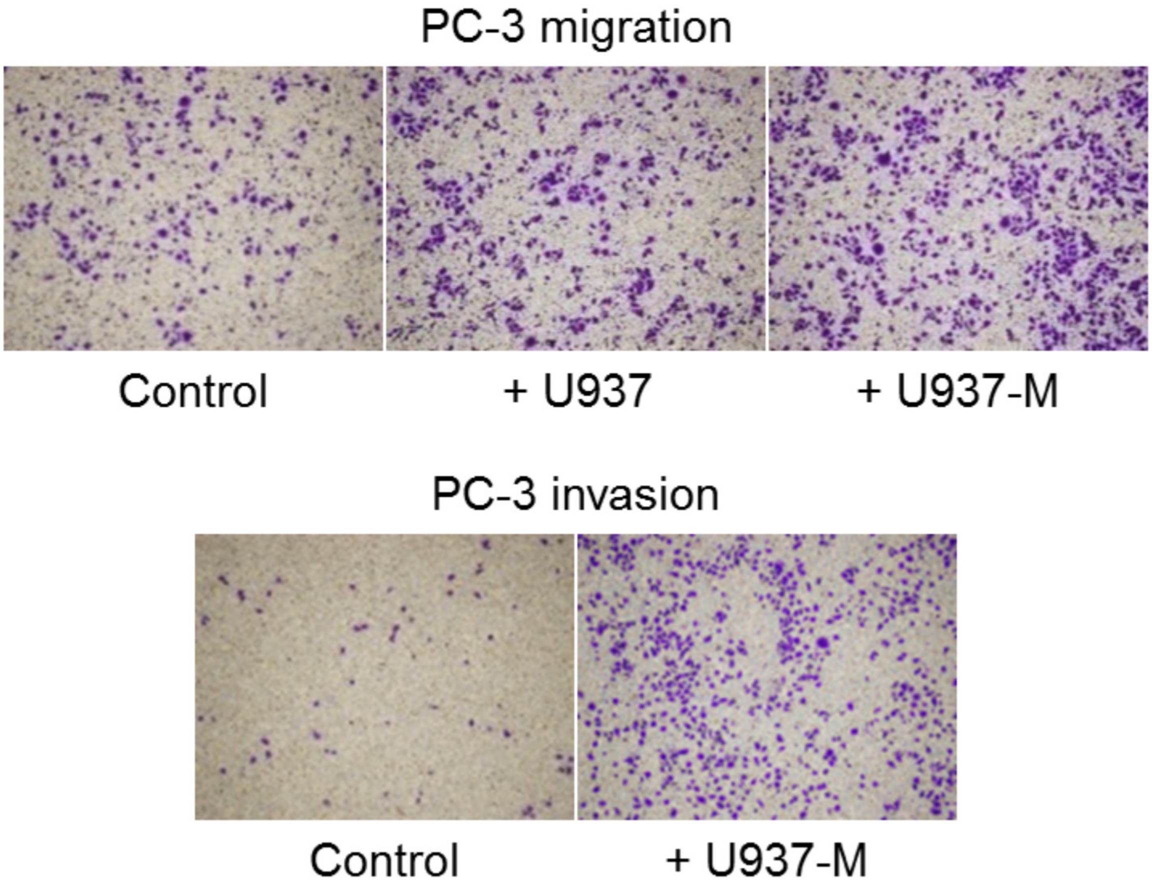


**Supplementary Figure 1: Pictures of PC-3 migration and invasion.** Representative pictures of migration and invasion assay are shown for better understanding the results intuitively. These pictures are corresponding to Figures 1B and C.

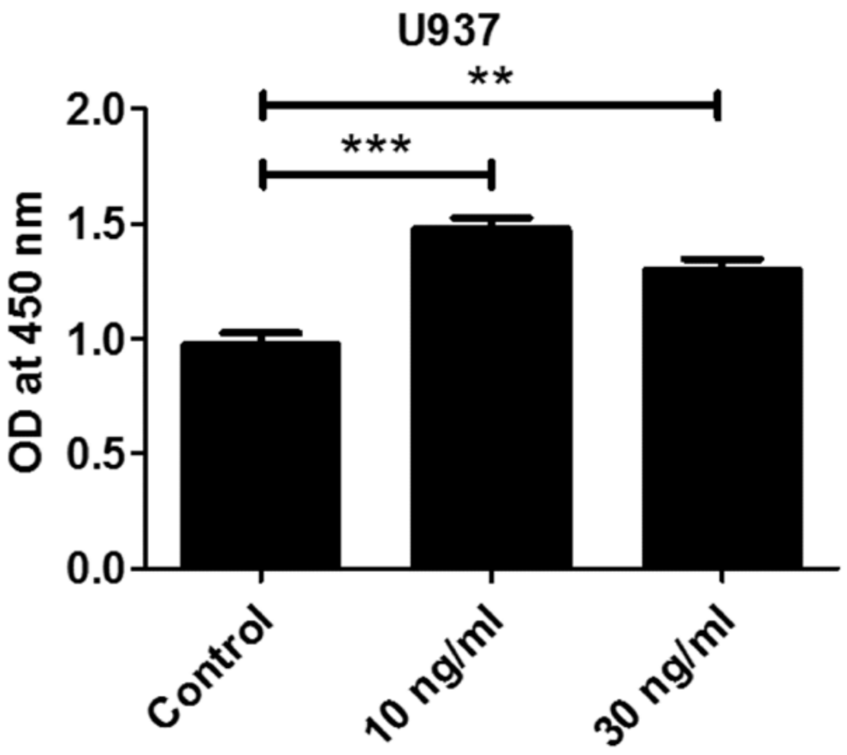


**Supplementary Figure 2: CCL2 promoted U937 cell migration.** The U937 cell migration assay is performed using HTS Transwell® 96-Well Permeable Supports with Barcode, Lid, and Receiver Plate (8.0-μm pore sizes, Corning). In the upper compartment, 1 × 105 cells in 100 μl of RPMI-1640 culture media supplemented with 0.1% FBS are placed, and the lower compartment is filled with 150 μl of RPMI-1640 containing 10% FBS with different concentrations of CCL2 (0–30 ng/ml). After incubation for 4 h, WST-1 reagent (Takara) is added to the lower compartment following the manufacturer's instructions, and the plates are maintained at 37°C for 110 min. Dye conversion is ascertained at 440 nm and corrected at 450 nm on a microplate reader. Statistical analysis is performed using Student's t-test. The data are presented as the mean ± SD. ** $p < 0.01$, *** $p < 0.001$.

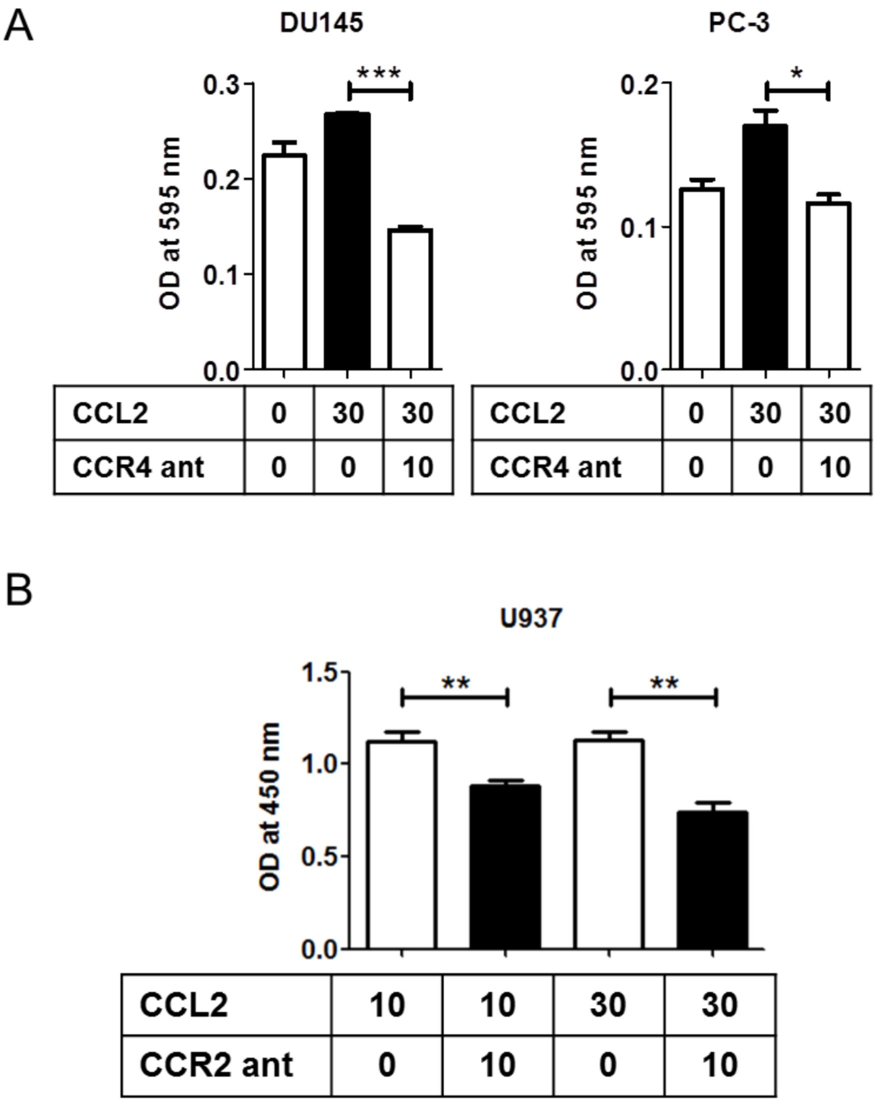


**Supplementary Figure 3: Blockade of CCR4 and CCR2 inhibited migration of prostate cancer cells and U937 cells, respectively. A.** Prostate cancer cells are incubated with CCR4 antagonist (CCR4 ant; 10 μg/ml) for 30 min and then incubated with CCL2 (30 ng/ml) for 24 h. The mean optical density (OD) value is read by a microreader at 595 nm. **B.** U937 cells are incubated with CCR2 antagonist (CCR2 ant; 0 or 10 μg/ml) for 30 min in RPMI-1640 culture media supplemented with 0.1% FBS at room temperature. The procedures are as for the U937 migration assay in Supplementary Figure 1. The data are presented as the mean ± SD. All experiments are performed in triplicate and mean values are shown. * $p < 0.05$, ** $p < 0.01$, *** $p < 0.001$.

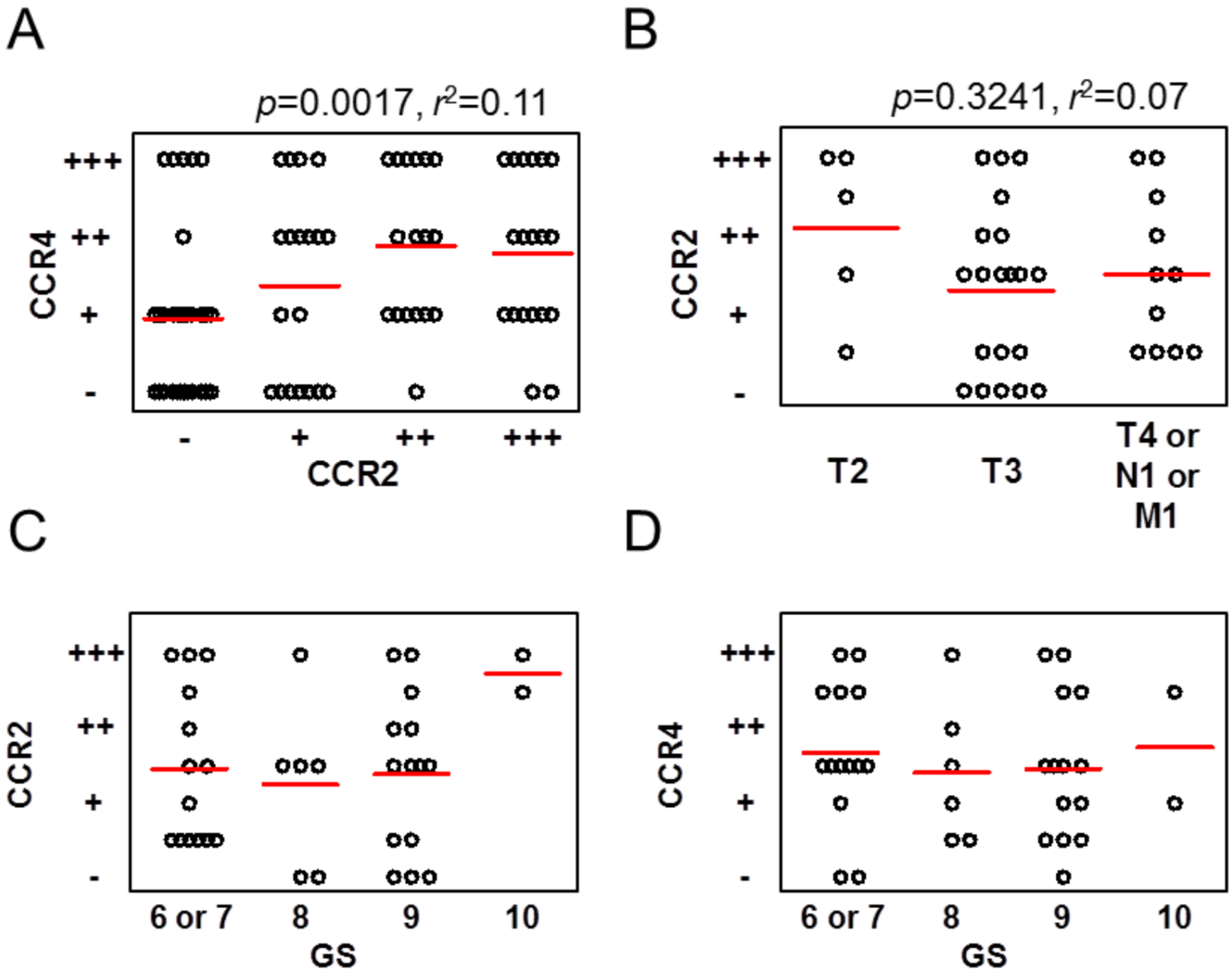


**Supplementary Figure 4: IHC staining of CCR4 and CCR2 is performed in human normal prostate tissues and prostate cancer tissues. A.** The correlation between the staining intensities of CCR2 and CCR4 in each specimen is analyzed using Pearson's correlation coefficient test. **B.** The CCR2 staining intensity is analyzed according to progression of TNM stage. **C, D.** The CCR2 (C) and CCR4 (D) staining intensities are analyzed according to Gleason score (GS). One-way ANOVA analysis is performed. Bars indicate the mean of each group.

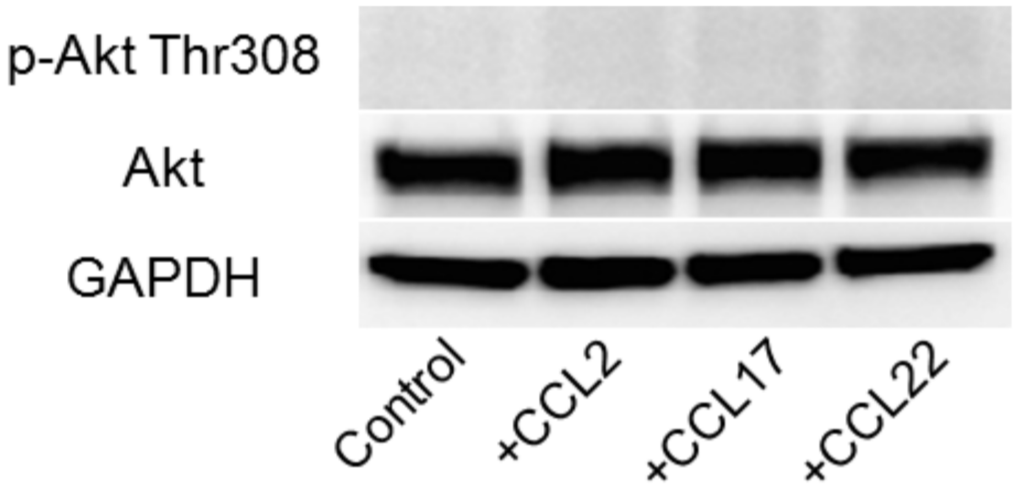


**Supplementary Figure 5: Phosphorylation of Akt Thr308.** Phosphorylation of Akt (Thr308) PC-3 cells is assayed by western blot at 30 min after CCL2, CCL17, or CCL22 stimulation. Adjustments of brightness, contrast, and size are applied to the whole image of western blot-based analyses without elimination of any information present in the original, including backgrounds.

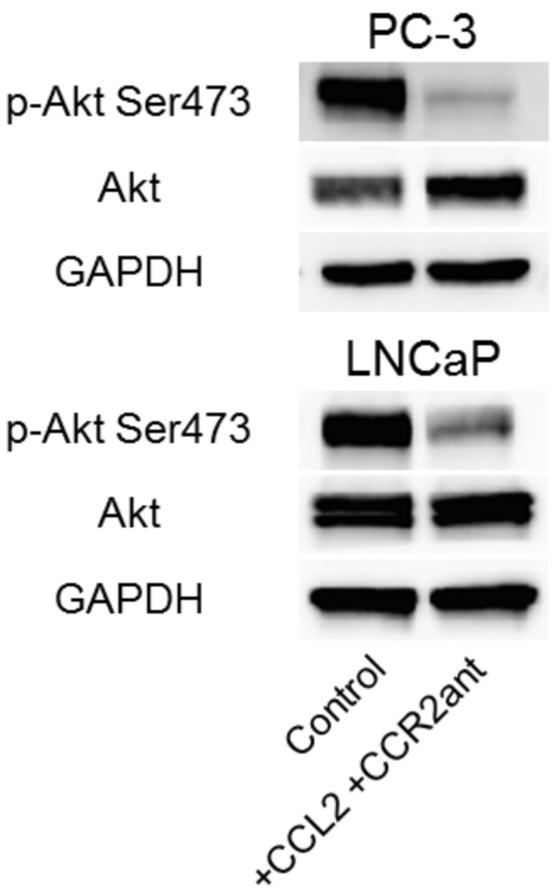


**Supplementary Figure 6: CCR2 antagonist blocked CCL2-induced phosphorylation of Akt.** Phosphorylation of Akt proteins in prostate cancer cells are assayed by western blot at 30 min after CCR2 antagonist treatment with CCL2 stimulation. Adjustments of brightness, contrast, and size are applied to the whole image of western blot-based analyses without elimination of any information present in the original, including backgrounds.

**Supplementary Table 1: Background of TMA patients**

| | | Normal | Cancer |
|---|---|---|---|
| n | | 8 | 36 |
| Median age, year | | 67 (44-70) | 66 (44-75) |
| Median PSA, ng/ml | | | 17.2 (0.5-161)† |
| T stage | 2 | | 8 |
| | 3 | | 26 |
| | 4 | | 2 |
| N stage | 0 | | 35 |
| | 1 | | 1 |
| M stage | 0 | | 24 |
| | 1 | | 8 |
| Gleason score | 6 | | 1 |
| | 7 | | 13 |
| | 8 | | 6 |
| | 9 | | 14 |
| | 10 | | 2 |

TMA, tissue microarray; PSA, prostate-specific antigen. †Data are not available in five patients.

**Supplementary Table 2: The sequences of primers**

| Gene | | Sequence |
|---|---|---|
| GAPDH | Forward | 5'-TCC ACC ACC CTG TTG GTG TA-3' |
| | Reverse | 5'-GAC CAC AGT CCA TGC CAT CA-3' |
| CCR2 | Forward | 5'-CTG TCC ACA TCT CGT TCT CGG TTT A-3' |
| | Reverse | 5'-CCC AAA GAC CCA CTC ATT TGC AGC-3' |
| CCR4 | Forward | 5'-AAG AAG AAC AAG GCG GTG AAG AAT G-3' |
| | Reverse | 5'-AGG CCC CTG CAG GTT TTG AAG-3' |
